# Charing Cross and Other Hospitals

**Published:** 1888-12-01

**Authors:** 


### Charing Cross and Other Hospitals.

By the Right Hon. the Earl of Derby, who presided at the Festival Dinner of the Charing Cross Hospital held last week.

It is not easy to argue a case when there is nothing or next to nothing to argue against. If anybody would be kind enough to dispute the usefulness of hospitals the case would be different, and there would then be some possibility of discussion; but it is scarcely practicable to insist at length on a proposition which, I think, everybody is agreed upon. I will not therefore dwell upon the customary commonplaces. We all know that hospitals are less liable to abuse than any other form of charity, because while distress very often is, and always may be, simulated, illness cannot always be so simulated, and, in point of fact, in very few cases is the attempt ever made. We know, further, that the function of the hospital is not limited to the relief of the individual sufferer, but that it is a school of medical science—a school of the most practical kind, by the existence of which none benefit more than those who are never likely to enter the door of any public institution except as visitors. We know, again, that the functions performed by our great public hospitals are absolutely indispensable. The want which they meet must be met by some means; and if the liberality of private persons did not supply that want, another heavy burden would be added to our existing rates and taxes, which, most of us think, are heavy enough already. We know lastly, and it is very well that it should be so, many persons who feel strongly their duties to society in general, and especially to the poorer classes of society, but who, from habits and occupation, can only assist those poorer classes by money contributions, which they have to leave to be distributed by others. Now to such persons our great hospitals supply exactly what they want—an investment, so to speak, for their charity—an investment which is perfectly safe, and into which they can put their money without the slightest fear that by what they give they are doing more harm than good, a result which, in indirect philanthropy, is exceedingly likely to occur.

Now, there is one difficulty connected with the administration of hospitals through which, perhaps, we do not quite see our way, and that is how to prevent the gratuitous treatment which hospitals give from being used and abused by those who can perfectly afford to pay. That is a question often raised, but if you think that I am going to provide you with the satisfactory answer to it, I am afraid you will be disappointed. It may be met in part by the reception of paying patients, and that is now done, I am informed, in some hospitals; but not in the Charing Cross Hospital. Farther, it may be met by a reference to those subscribers who send their dependents to be taken care of, and who are morally bound to see that those dependents do not, as occupying beds, exclude the needy and those who have no one to look after them. Provident dispensaries will also do something. I cannot enlarge upon the subject here, but if this had been a more suitable time or place, there is a good deal to be said as to the possibility and as to the expediency of extending the system of provident dispensaries, and of working it in connexion with our great hospitals. That is a subject for consideration and action hereafter, and it is not one in connexion with which I am prepared to propose anything definite now. But, after all, our greatest reliance must be upon that strong in-born spirit of independence, which even all our lavish and sometimes rather indiscriminate charity has not destroyed, or to any considerable extent enfeebled. Gentlemen, I should think I was wasting your time if I detained you by any notice of attempts which have been made lately to disparage these noble institutions by representing those who manage them as regarding their patients only in the light of subjects for scientific experiment. How utterly remote from the truth are charges of that kind you do not want to be told. They are not meant to be taken seriously; they are not inspired by sympathy for suffering; and if they have any serious meaning, they are only the outbreak of that jealousy and alarm with which in some quarters the progress of the scientific spirit is always viewed. So far as I can speak from observation, I should say without hesitation, that of al professional persons medical men are those who give the largest portion of their time, unrewarded and often unthanked, to the relief of human misery. They often sacrifice theii own health to that of the patients whom they are attending, and, knowing that they have entered a service in which the prizes are comparatively few, they work in it ungrudgingly, often ill paid, generally obscure, and rewarded only by the sense of public usefulness, and by a feeling of public duty.

Leaving these generalities, let me mention a few details with which I have been supplied, as to the present state of the Charing Cross Hospital. I am glad to say our latest reports speak in confident and even-sanguine tones as to its prospects and future. Three years ago a festival like this was held, and the wants of the hospital were then stated to be a separate children's ward for medical and surgical cases, a separate accident ward, and improvements in the hospital theatre. All these wants have been supplied. The value of the separate accident ward is in the facility which it gives for the admission of accident cases at any time of the day and night without disturbance to other patients. I am glad to be able to say that this ward has been supplied entirely by a widow lady, and its value and importance in such an important and central part of London as Charing Cross are obvious. In the present year 8,800 cases due to accidents were treated, making an average of about 28 a-day. On the one day of the riots in Trafalgar Square 75 cases were received in three hours; and on Jubilee Day, when the people were perfectly well behaved, but where it was impossible with such large crowds to avoid accidents, 95 cases of accident were brought in. The theatre is now one of the best in existence, and other improvements of detail have been made with which I shall not trouble you. I am happy to be able to tell you, and I confess it strikes me with a feeling of surprise, that the last three years have been the most prosperous in the whole history of this hospital. In those three years there have been treated 4,800 in-patients, and more than 66,000 out-patients. The number of patients admitted in the present year up to date has exceeded that of any previous year, and out of 30 beds which hard times compelled us to close ten years ago, 25 have been opened again. I will not trouble you with figures of income and expenditure, but you will be glad to hear that the income of the hospital has sufficed to meet the general outlay, to pay for the necessary building of which I have spoken, and to put by £6,000 against bad times. I doubt if any hospital, or, indeed, any charitable institution, in London can show an equally satisfactory record. The fixed and certain income of the hospital is, and has long been more than £6,000 short of the normal outlay, and yet there has never been a deficit. The public has been expected to make the deficiency good, and has always come up to the mark. We have, however, fresh work on hand. The medical school, I am told, absolutely requires extension, and for this simple and satisfactory reason, that the number of students has doubled in the last three years. The nursing establishment is to be taken in hand by the hospital directly. For twenty years past it has been managed by arrangement or contract with a nursing body or association outside, but of late it has been thought desirable to have it under direct control, and arrangements with that view are being made. It is hoped that the permanent increase of cost will not be large, and perhaps there will be none; but an increased amount of accommodation is an absolute necessity. The new buildings required, together with the grounds, will probably cost £20,000, and the committee speak confidently of getting that sum before the next triennial meeting. Our last requirement is one the advantage of which will be very obvious. It is a convalescent home. If a patient in the convalescent stage is kept in the hospital, he is evidently occupying a bed which is needed for some case of more severe suffering. If, on the other hand, he is sent back half recovered to what is in too many cases a very miserable home, his progress towards recovery is possibly impeded, and the good he gained in the hospital is lost again. These evils would be obviated, as you can clearly see, if we have a convalescent home. These are our wants, and my friends who know more than I do of what the public has done for them in the past, and may be reasonably expected to do in the future, are confident that they will be supplied. There is one case of munificence which I desire to mention. As you all know, there is no family which has been more helpful to us for many years past than one which is celebrated in connexion with the daily press —I mean the name of Levy. At the last triennial festival the amount of their donation was £4,000, and I am happy to say that we have received from Miss Matilda Levy, in memory of her late father, a gift of £1,000 additional. I think I may congratulate you upon the attendance here to-night, and upon the amount of subscriptions which you will shortly hear mentioned. The amount would be creditable at any time, and it is doubly so now, when, as we know, the incomes of the richer classes have fallen off, from landed and other depressions, and when the funds of so many charitable institutions have suffered in proportion.

